# Efficacy of intensive antiemetic therapy including olanzapine in multiple myeloma patients treated with high-dose melphalan with autologous stem cell transplantation

**DOI:** 10.1007/s00520-025-09839-2

**Published:** 2025-08-11

**Authors:** Junpei Kato, Masashi Uchida, Masayuki Ishikawa, Shinya Tatsuta, Takeshi Yoshimi, Kota Ishida, Osamu Hosoya, Nobuhiro Tsukada, Tadao Ishida, Yuki Shiko, Yohei Kawasaki, Shingo Yamazaki, Itsuko Ishii

**Affiliations:** 1 Department of Pharmacy, Japanese Red Cross Medical Center, Tokyo, Japan; 2 Graduate School of Pharmaceutical Sciences, Chiba University, Chiba, Japan; 3 Division of Pharmacy, Chiba University Hospital, 1-8-1 Inohana, Chuo-ku, Chiba, 260-8677 Japan; 4 Department of Hematology, Japanese Red Cross Medical Center, Tokyo, Japan; 5 Department of Biostatistics, Graduate School of Medicine, Saitama Medical University, Saitama, Japan

**Keywords:** Olanzapine, Chemotherapy-induced nausea and vomiting, High-dose melphalan, Autologous stem cell transplantation multiple myeloma

## Abstract

**Purpose:**

Conditioning with high-dose melphalan (MEL) followed by autologous stem cell transplantation (ASCT) is the standard treatment for multiple myeloma (MM). The optimal regimen to prevent chemotherapy-induced nausea and vomiting (CINV) is unclear. We aimed to retrospectively evaluate the antiemetic effect and safety of a four-drug intensive regimen including olanzapine (OLA) on CINV in MM patients receiving MEL/ASCT.

**Methods:**

MEL (200 mg/m^2^) was administered on day 1, followed by ASCT on day 3. Patients were classified into the standard group (palonosetron and dexamethasone on day 1, and aprepitant on day 1–3), and the intensive antiemetic regimen (IAR) group (palonosetron on day 1, dexamethasone on day 1–2, and aprepitant, and OLA on day 1–5). The primary endpoint was defined as no vomiting and no rescue medications (complete response) in the delayed phase (day 2–5).

**Results:**

There were no significant differences in baseline characteristics between the OLA (n = 68) and standard (n = 54) groups. The complete response rate in the IAR group was significantly higher in the delayed phase (52.9% vs. 31.4%, *p* < 0.05). Multivariate analysis revealed that the IAR was associated with the complete response rate (OR, 2.34; 95% CI, 1.09–5.00; *p* = 0.028). The incidence of nausea (grade 3) in the delayed phase was lower in the IAR group (44.1% vs. 75.9%, *p* < 0.001).

**Conclusion:**

The four-drug intensive regimen including OLA may improve the antiemetic effect on delayed CINV while also ensuring safety in MM patients undergoing MEL/ASCT.

## Introduction

Multiple myeloma (MM) is a malignancy of plasma cells derived from B cells. For patients newly diagnosed with MM, a melphalan (MEL) conditioning regimen followed by autologous stem cell transplantation (ASCT) has shown superior outcomes in terms of achieving a complete response and longer progression-free survival compared with chemotherapy alone [1, 2].

Chemotherapy-induced nausea and vomiting (CINV) is one of the most significant non-hematologic adverse effects of high-dose MEL chemotherapy, and it frequently compromises the patient’s quality of life (QOL) [3, 4]. The emetogenic risk of antineoplastic agents and individual patient risk factors have been linked to the incidence and severity of CINV [5]. Several reports have shown that the incidence of CINV is higher in the delayed phase (onset within 24 to 120 h after chemotherapy administration) than in the acute phase (onset within 24 h after chemotherapy administration) in MM patients undergoing MEL/ASCT, and the control of CINV in the delayed phase is inadequate [6, 7]. Although no consensus has been reached on the emetogenic potential of high-dose MEL, it has recently been reported to be a high-risk agent in various guidelines [5, 8, 9].

Studies have shown that a three-drug regimen including aprepitant (APR), a serotonin (5-HT_3_)-receptor antagonist, and dexamethasone (DEX) is more effective than a two-drug regimen for CINV in MM patients undergoing MEL/ASCT [6, 10]. Several studies have demonstrated that a four-drug regimen including OLA has a prophylactic antiemetic effect on solid malignancies [11, 12]. The FOND-O study was a randomized, double-blind, placebo-controlled clinical trial that evaluated the efficacy of a four-drug regimen including OLA compared with a three-drug regimen for patients receiving highly emetogenic chemotherapy (HEC) for hematologic malignancies or HSCT [13]. The addition of OLA to the three-drug regimen improved clinically relevant CINV outcomes, defined as a complete response (CR; no emesis and minimal nausea) in the delayed phase. Moreover, a subgroup analysis for patients with ASCT (i.e., MM patients treated with MEL [n = 25] and non-Hodgkin’s lymphoma patients treated with BEAM [carmustine, etoposide, cytarabine, melphalan; n = 19]) revealed that administration of the four-drug regimen significantly improved the CR rate in the delayed phase. Based on these findings, some antiemesis guidelines recommend a four-drug regimen including OLA for HSCT patients undergoing HEC conditioning regimens [5, 8, 9].

Nevertheless, there are currently no reports documenting the antiemetic effect of four-drug regimens including OLA in Japanese patients with MM undergoing MEL/ASCT. In addition, most studies assessing CINV in MM patients undergoing MEL/ASCT, including the FOND-O study, have used an observation period of 5 days [6, 7, 13, 14]. Recent studies have shown that approximately 20% of patients experience CINV beyond 120 h (5 days) after the administration of chemotherapy [15]. Some studies recommend extending the observation period for assessing CINV to 168 h (7 days) [16, 17]. Moreover, high-dose MEL can be administered in 1 day (200 mg/m^2^/day) or over 2 days (100 mg/m^2^/day) before ASCT [4, 18, 19]. Parmar et al. found that high-dose MEL given over 2 days significantly increased the frequency of grade ≥ 3 mucositis relative to the 1-day regimen [20]. The FOND-O study did not specify the method of administration of MEL; therefore, the antiemetic effect of the four-drug regimen including OLA and the 1-day high-dose MEL regimen, on CINV remains unknown.

Therefore, we aimed to evaluate the antiemetic effect and safety of a four-drug intensive regimen including OLA on CINV for 7 days after the administration of chemotherapy in Japanese MM patients treated with 1-day high-dose MEL/ASCT.

## Methods

### Patients

This single-center retrospective study was conducted at the Japanese Red Cross Medical Center (Tokyo, Japan). The study included MM patients who underwent MEL/ASCT at the center between July 2020 and March 2024. The exclusion criteria were as follows: patients who experienced nausea and vomiting within 12 h prior to starting high-dose MEL, patients who used antiemetic drugs within 24 h prior to starting high-dose MEL, patients who developed complications with gastrointestinal symptoms during the observation period (e.g., ileus, pancreatitis), and patients with pre-existing type 2 diabetes who could not receive OLA as it is contraindicated in Japan.

### Treatment

MEL (200 mg/m^2^, i.v.) was administered on day 1, followed by ASCT on day 3. MEL dose adjustments were made at the physician’s discretion in consideration of the patient’s overall physical condition including renal, hepatic, and cardiac function and other factors. Patients who received palonosetron (PAL, 0.75 mg i.v. on day 1), dexamethasone (DEX, 6.6 mg i.v. on day 1), and aprepitant (APR, 125 mg p.o. on day 1 and 80 mg p.o. on days 2 and 3) as antiemetics were designated as the standard group (July 2020 to March 2021). Patients receiving PAL (0.75 mg i.v. on day 1), DEX (9.9 mg i.v. on day 1 and 6.6 mg i.v. on day 2), APR (125 mg p.o. on day 1 and 80 mg p.o. on days 2–5), and OLA (5 mg p.o. on days 1–5) were designated as the IAR group (April 2021 to March 2024) (Table 1). The PAL dose was the standard approved dose for patients aged ≥ 18 years in Japan. The DEX dose was lower than the standard dose for HEC due to the risk of infection after ASCT. On day 1, APR was administered 1–1.5 h before the administration of MEL, while PAL and DEX were given 30 min before the administration of MEL. OLA was administered after dinner. Rescue medication (metoclopramide [5 mg p.o.] or metoclopramide [10 mg i.v.]) for breakthrough CINV was provided at the physician’s discretion.

**Table 1:** Treatment schedule

	MEL 200 mg/m^2^		ASCT		
Regimen	day 1	day 2	day 3	day 4	day 5
IAR group					
Aprepitant, mg	125	80	80	80	80
Palonosetron, mg	0.75				
Dexamethasone, mg	9.9	6.6			
Olanzapine, mg	5	5	5	5	5
Standard group					
Aprepitant, mg	125	80	80		
Palonosetron, mg	0.75				
Dexamethasone, mg	6.6				

*MEL* melphalan, *ASCT* autologous stem cell transplantation, *IAR* intensive antiemetic regimen

### Data Collection

The observation period was from day 1 to 7. We further defined the observation period as follows: acute phase (day 1 of MEL administration), delayed phase (day 2 to 5), beyond delayed phase (day 6 to 7), and all phases (day 1 to 7). Data were obtained through a retrospective review of electronic medical records. Patient background characteristics included age, sex, MM type, Revised International Staging System (R-ISS), previous treatments, opioid use, history of ASCT, the doses of MEL and dimethyl sulfoxide (DMSO), and laboratory data (aspartate aminotransferase [AST], alanine aminotransferase [ALT], total bilirubin [T-Bil], albumin [Alb], serum creatinine [Scr] measured by an enzymatic method, serum sodium [Na], serum chloride [Cl], serum potassium [K], serum calcium [Ca], corrected by serum albumin). Estimates of creatinine clearance (CrCl) were based on the Cockcroft-Gault formula using actual total body weight. Emesis was defined as observed vomiting and was assessed based on a review of electronic medical records recorded by nursing staff. Nausea (grade 3) was defined based on the use of total parenteral nutrition (TPN) according to the Common Terminology Criteria for Adverse Events (CTCAE) v5.0. The use of rescue medication and drug compliance were assessed through a review of the medication administration records. Adverse events such as constipation, diarrhea, hyperglycemia, and hiccup were assessed according to CTCAE v5.0.

### Study Endpoints

The primary endpoint was the CR (no emesis and no use of rescue medication) rate during the delayed phase. The secondary endpoints were the CR rate during the acute phase, the beyond delayed phase, and all phases, the identification of factors associated with the CR rate, the incidence of nausea (grade 3) in each phase, and the overall incidence of treatment-related adverse events during all phases.

### Statistical analysis

Demographic and descriptive data were compared between the IAR and standard groups using the Mann–Whitney *U* test for continuous data and the chi-square test or Fisher’s exact test for categorical data. The chi-square test was used to analyze the CR rate in each phase. Univariate logistic regression analysis and multivariate logistic regression analysis with a hierarchical backward elimination method were conducted to identify independent predictors of the CR rate. A *p-*value of 0.1 was used as the threshold for backward selection. The independent variables included the antiemetic regimen (standard group as a reference), sex (female as a reference), age, CrCl, MEL dose (%), and DMSO dose (g/kg) in cryopreserved hematopoietic stem cell products. In HSCT, transfusion itself is known to cause adverse events such as nausea and vomiting, which have been attributed to the toxicity of DMSO in cryopreserved hematopoietic stem cell products [21]. The chi-square test or Fisher’s exact test was used to analyze the secondary endpoint of the incidence of nausea (grade 3) in each phase. The incidence of treatment-related adverse events was categorized as any grade or grade ≥ 3, and analyzed using the chi-square test or Fisher’s exact test. No adjustment was made for multiple comparisons across multiple endpoints. Analyses were performed with SPSS Statistics (ver. 29.0, IBM Corp, Armonk, NY). Statistical significance was defined as *p* < 0.05.

## Results

### Efficacy analysis

A total of 134 patients with MM undergoing high-dose MEL therapy and ASCT were screened. The following patients were excluded from the study: 4 patients from the standard group who deviated from the antiemetic protocol, 7 patients from the IAR group who had type 2 diabetes, and 1 patient who developed pancreatitis during the study period (making it difficult to assess CINV). Consequently, a total of 122 patients were enrolled (IAR group, n = 68; standard group, n = 54). There were no significant differences in patient background between the two groups (Table 2).

**Table 2:** Demographic and clinical characteristics

	IAR group (n = 68)	Standard group (n = 54)	*P* value
*Age*			
Median (years)	57 [25–68]	56.5 [39–70]	0.640^a)^
≤ 65 years	59 (86.7)	47 (87)	0.802 ^b)^
*Sex*			
Male	47 (69.1)	29 (53.7)	0.288^b)^
Female	21 (30.9)	25 (46.3)	
*Disease type*			0.650^b)^
IgG	30 (44.1)	25 (46.3)	
IgA	11 (16.1)	8 (14.8)	
BJP	19 (27.9)	18 (33.3)	
Others	8 (8.0)	3 (5.5)	
*International Staging System*			0.559^b)^
R-ISSI	13 (19.1)	13 (24.1)	
R-ISSII	33 (48.5)	23 (42.6)	
R-ISSIII	14 (20.5)	8 (14.8)	
Unknown	8 (11.7)	10 (18.5)	
Prior myeloma treatment	2 [1–13]	2 [1–7]	0.626^a)^
Opioid use	13 (19.1)	9 (16.7)	0.727^b)^
History of ASCT	11 (16.1)	9 (16.7)	0.446^b)^
AST	17 [8–37]	16 [9–34]	0.484^a)^
ALT	16 [4–91]	15 [5–68]	0.301 ^a)^
T-Bil	0.6 [0.2–1.7]	0.5 [0.2–1.4]	0.297 ^a)^
ALB	3.9 [2.7–4.9]	3.9 [2.5–4.9]	0.772 ^a)^
Scr	0.79 [0.44–2.36]	0.76 [0.4–2.68]	0.969 ^a)^
CrCl (mL/min)	85.2 [19.5–184]	83.2 [24.7–136]	0.628 ^a)^
MEL (%)	100 [70–100]	100 [63–100]	0.070 ^a)^
*MEL dose* MEL 200 mg/m^2^ MEL < 200 mg/m^2^	63 (92.6) 5 (7.4)	51 (94.4) 3 (5.6)	1.000 ^b)^
DMSO (g/kg)	0.16 [0.03–0.72]	0.21 [0.06–0.61]	0.682 ^a)^

Data are shown as the median [range] or n (%). *MEL* (%) indicates the percentage of the 200 mg/m^2^ dose. a) Mann–Whitney *U* test, b) Fisher’s exact test. *IAR* intensive antiemetic regimen, *BJP* Bence Jones protein, *R-ISS* revised-international staging system, *ASCT* autologous stem cell transplantation, *AST* aspartate aminotransferase, *ALT* alanine aminotransferase, *T-Bil* total bilirubin, *ALB* albumin, *Scr* serum creatinine, *CrCl* creatinine clearance, *MEL* melphalan, *DMSO* dimethyl sulfoxide

**Fig. 1:**
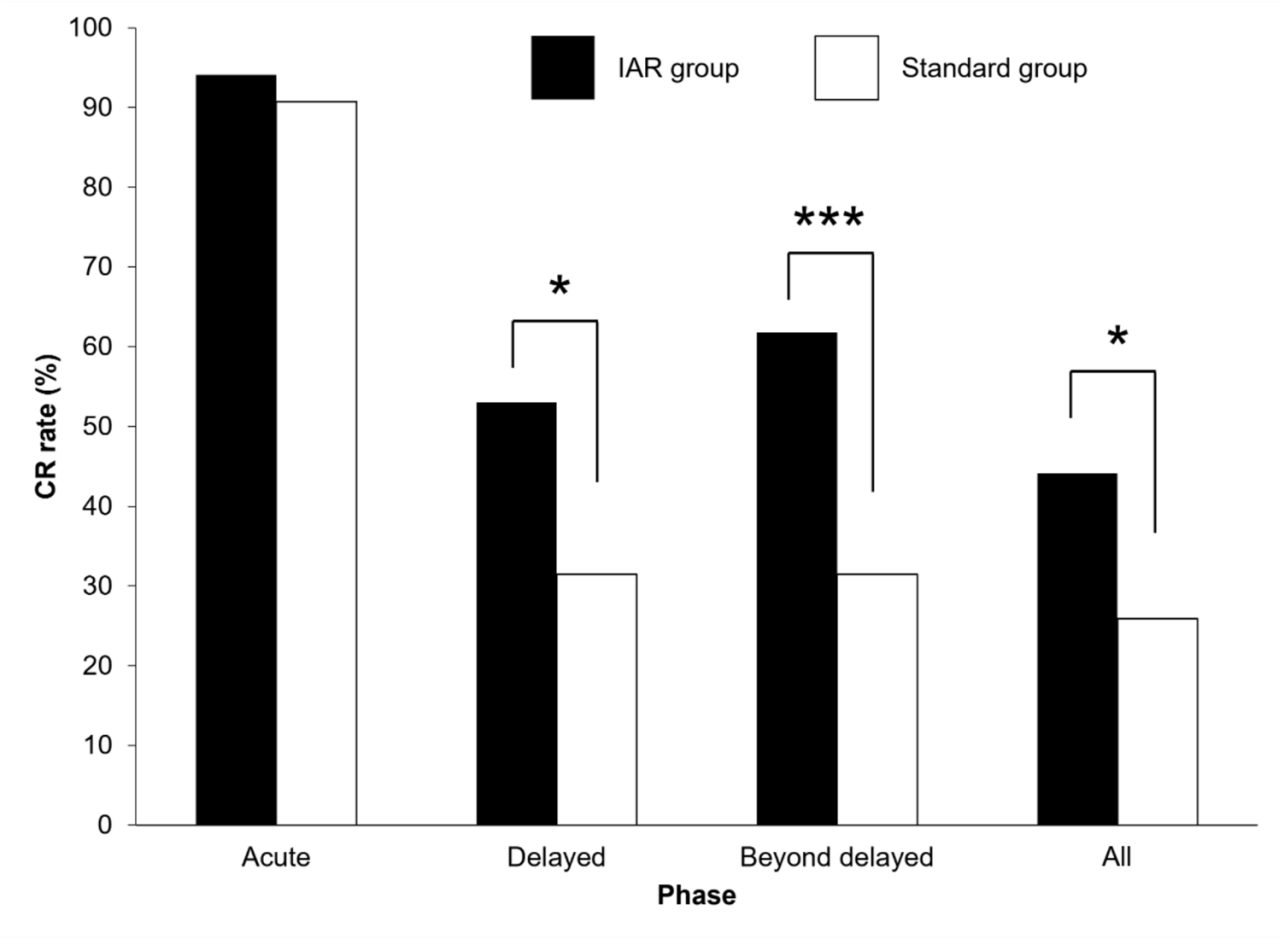
CR rate during each phase. Statistical analysis was performed using Fisher’s exact test. * *p* < 0.05, *** *p* < 0.001. CR, complete response; IAR, intensive antiemetic regimen

The primary endpoint, the CR rate in the delayed phase, was significantly higher in the IAR group than in the standard group (52.9% vs. 31.4%, *p* < 0.05). There was no significant difference between the two groups in the CR rate during the acute phase (94.1% vs. 90.7%). In the beyond delayed phase, the CR rate was significantly higher in the IAR group than in the standard group (61.8% vs. 31.4%, *p* < 0.001). In all phases, the CR rate was significantly higher in the IAR group than in the standard group (44.1% vs. 25.9%, *p* = 0.038) (Fig. 1).

Univariate and multivariate logistic regression analyses were conducted to identify factors associated with the CR rate (Table 3). According to the multivariate analysis, age was associated with the CR rate in the acute phase (adjusted odds ratio [aOR], 1.07; 95% confidence interval [CI], 1.00–1.15; *p* = 0.049), the IAR was associated with the CR rate in the delayed phase (aOR, 2.34; 95% CI, 1.10–5.00; *p* = 0.028), the IAR (aOR, 3.42; 95% CI, 1.58–7.42; *p* = 0.002) and CrCl (aOR, 1.01; 95%CI, 1.02–1.03; *p* = 0.042) were associated with the CR rate in the beyond delayed phase, and sex (aOR, 2.88; 95% CI, 1.26–6.57; *p* = 0.012) was associated with the CR rate in all phases. No other factors were associated with the CR rate (Table 3).

**Table 3:** Identification of factors associated with CR rates during each phase

	Acute						Delayed							Beyond delayed						All											
	Crude			Adjusted			Crude			Adjusted				Crude			Adjusted			Crude						Adjusted					
Variable	OR	95% CI	*P* value	OR	95% CI	*P* value	OR	95% CI	*P* value		OR	95% CI	*P* value	OR	95% CI	*P* value	OR	95% CI	*P* value	OR		95% CI		*P* value		OR		95% CI		*P* value	
IAR group	0.48	0.42–6.40	0.482	―	―	―	2.45	1.16–5.16	0.019		2.34	1.10–5.00	0.028	3.52	1.65–7.48	0.001	3.42	1.58–7.42	0.002	2.26		1.04–4.89		0.039		2.14		0.97–4.73		0.061	
Sex (male)	1.89	0.48–7.41	0.362	―	―	―	2.25	1.06–4.77	0.035		2.14	0.99–4.61	0.053	2.04	0.98–4.26	0.058	―	―	―	3.00		1.33–6.77		0.008		2.88		1.26–6.57		0.012	
Age (years)	1.08	1.01–1.16	0.026	1.07	1.00–1.15	0.049	1.01	0.97–1.05	0.826		―	―	―	0.99	0.95–1.03	0.517	―	―	―	0.98		0.94–1.02		0.398		―		―		―	
CrCl (mL/min)	0.99	0.97–1.01	0.234	―	―	―	1.01	0.99–1.02	0.34		―	―	―	1.01	1.00–1.02	0.115	1.02	1.00–1.03	0.042	1.01		1.00–1.02		0.196		―		―		―	
MEL (%)	0.88	0.58–1.32	0.531	―	―	―	0.98	0.93–1.02	0.31		―	―	―	0.97	0.92–1.02	0.207	0.95	0.90–1.00	0.063	0.97		0.92–1.02		0.178		―		―		―	
DMSO (g/kg)	―	―	―	―	―	―	0.65	0.05–8.00	0.733		―	―	―	0.21	0.02–2.72	0.232	―	―	―	0.15		0.01–2.53		0.191		―		―		―	

*OR* odds ratio, 95% *Cl* 95% confidence interval, *aOR* adjusted odds ratio, *IAR* intensive antiemetic regimen, *CrCl* creatinine clearance, *MEL* melphalan, *DMSO* dimethyl sulfoxide

**Fig. 2:**
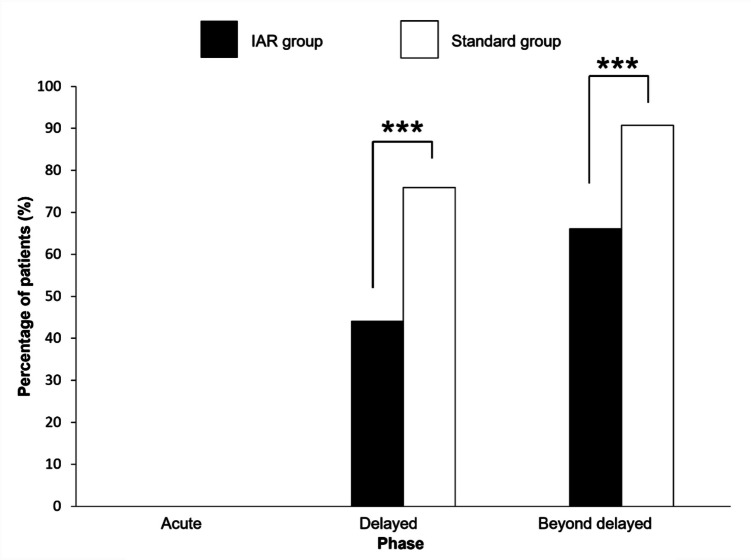
Incidence of grade 3 nausea during each phase. The incidence of grade 3 nausea was assessed based on the use of total parenteral nutrition in each phase. Statistical analysis was performed using Fisher’s exact test. *** *p* < 0.001. IAR, intensive antiemetic regimen

The secondary endpoint, the incidence of nausea (grade 3) was assessed by the percentage of patients treated with TPN (Fig. 2). TPN was initiated at the physician’s discretion for patients with decreased appetite due to nausea and vomiting. No patients showed a decrease in food intake due to stomatitis, sore throat, or dysphagia during the observation period. No cases of nausea were observed in the acute phase. The incidence of nausea during the delayed phase (44.1% vs. 75.9%,* p* < 0.001), and the beyond delayed phase (66.2% vs. 90.7%, *p* < 0.001) was significantly lower in the IAR group than in the standard group.

### Safety analysis

The treatment-related adverse events in all phases were evaluated (Table 4). The most common adverse event was any-grade diarrhea, which occurred in 74.0% of the patients in the IAR group and 72.2% of the patients in the standard group. However, the incidence of grade ≥ 3 diarrhea was significantly lower in the IAR group than in the standard group (2.9% vs. 14.8%, *p* = 0.022). The incidence of any-grade constipation was significantly higher in the IAR group than in the standard group (43.0% vs. 22.2%, *p* = 0.018). There was no significant difference in hyperglycemia between the two groups. The incidence of any-grade hiccup was significantly higher in the IAR group than in the standard group (57.3% vs. 11.1%, *p* < 0.001), and 1 patient in the IAR group experienced grade 3 hiccup. There were no grade 4 adverse events or events that led to treatment discontinuation.

**Table 4:** Treatment-related adverse events

		IAR group (n: 68)	Standard group (n: 54)	*P* value
Constipation	Any grade	29 (43.0)	12 (22.2)	0.018
	Grade ≥ 3	0 (0)	0 (0)	-
Diarrhea	Any grade	50 (74.0)	39 (72.2)	0.872
	Grade ≥ 3	2 (2.9)	8 (14.8)	0.022
Hyperglycemia	Any grade	49 (72.0)	38 (70.4)	0.838
	Grade ≥ 3	0 (0)	0 (0)	-
Hiccup	Any grade	39 (57.3)	6 (11.1)	< 0.001
	Grade ≥ 3	1 (1.5)	0 (0)	1.000

Data are shown as n (%). Statistical analysis was performed using Fisher’s exact test. *IAR* intensive antiemetic regimen

## Discussion

CINV continues to pose a major challenge for MM patients undergoing MEL/ASCT. The high frequency of CINV during the delayed phase in patients undergoing MEL/ASCT often impairs their ability to eat food and take oral medications in the post-transplant period, which may prolong the hospital stay [6]. Furthermore, the incidence of CINV in MM patients undergoing MEL/ASCT is problematic not only in the delayed phase, but also in the beyond delayed phase [16]. Thus, in our study, the patients were observed for 7 days after the administration of MEL.

Some evidence indicates that OLA-including regimens may be superior to standard antiemetic therapy. A retrospective study found that OLA-based regimens were more effective than APR-based regimens in reducing acute and delayed CINV and the need for rescue medications [14]. The FOND-O study assessed the effect of the addition of OLA to a three-drug regimen (fosaprepitant, ondansetron, DEX) in patients undergoing ASCT. The study found a significant increase in the CR rate in patients who received a four-drug regimen including OLA [13]. However, conditioning regimens are not standardized (using MEL and BEAM) and the target diseases in the FOND-O study included not only MM but also non-Hodgkin’s lymphoma. Our study is unique not only for its long observation period but also for its focus on a single conditioning regimen and disease to obtain more specific results. In addition, OLA was administered at 10 mg once daily in the FOND-O study. Because the international standard dose of OLA (10 mg) induces significant drowsiness in the Japanese population, OLA (5 mg) is recommended for patients receiving HEC [12, 22]. Therefore, OLA was administered at a dose of 5 mg once daily in this study. Because the efficacy of a four-drug regimen for high-dose chemotherapy in patients undergoing ASCT has been demonstrated [13], we expect that the present IAR would be effective in other ethnic/racial populations. However, considering that the OLA dose of 5 mg is lower than that used in other ethnic/racial populations and considering that Asians are more prone to have CINV than other races [23], dose optimization of OLA and/or other antiemetics may be necessary when the present regimen is used in other ethnic/racial populations.

To our knowledge, this is the first study to investigate the control of CINV using a four-drug regimen including OLA in Japanese MM patients undergoing MEL/ASCT until the beyond delayed phase. Our findings show that the CR rates during the delayed and beyond delayed phases were significantly higher in the IAR group than in the standard group. Therefore, the use of a four-drug intensive regimen including OLA for the treatment of MM patients undergoing MEL/ASCT may improve delayed and beyond delayed CINV, help maintain nutrition and oral medication adherence after transplantation, and ultimately improve patient QOL and treatment outcomes. On the other hand, IAR was not significantly associated with the CR rate in all phases in the multivariate analysis. This may be because the difference in the CR rate between the two groups was small during the acute phase, and the effect of the IAR may be limited when evaluated in all phases.

It should be noted that the difference between the standard and IAR groups was not only the addition of OLA but also the intensification of treatment with DEX and APR. In the IAR group in the present study, the APR administration period was extended from 3 to 5 days, the DEX dose at day 1 was increased from 6.6 mg to 9.9 mg and a 6.6 mg dose was added to day 2 relative to the standard group. Thus, when assessing the antiemetic effectiveness during the delayed and beyond delayed phases, we must consider the effects of DEX and APR. The elimination half-life of DEX is about 5 h. However, drug-drug interactions with APR are known to reduce DEX clearance by approximately 50% [24, 25], suggesting that the effects of DEX may extend to about day 4. Previous studies have shown that a three-drug regimen in which APR was administered for 3 days improved delayed CINV in MM patients undergoing MEL/ASCT [6, 14], but there are no reports on the effects of a 5-day APR regimen. The effect of the 5-day APR regimen on CINV in the delayed and beyond delayed phases in MM patients undergoing MEL/ASCT is still unknown. The elimination half-life of APR is about 10 h [26], suggesting that the effect of APR could remain in the beyond delayed phase. OLA inhibits multiple neurotransmitter pathways known to be involved in CINV and may contribute to the improvement of CINV that cannot be suppressed by standard antiemetic therapy [27, 28]. The elimination half-life of OLA is about 33 h [29], which is longer than that of APR and DEX, suggesting that the antiemetic effect observed in the beyond delayed phase could be mainly due to OLA.

Multivariate analysis identified that in addition to antiemetic therapy, age (in the acute phase), CrCl (in the beyond delayed phase) and sex (in all phases) were significantly related to the CR rate. Previous studies have identified age (younger) and sex (female) as factors related to CINV [30, 31]. The relationship between age, sex, and the CR rate is consistent with previous reports. Several studies have investigated the pharmacokinetics of MEL in MM patients with renal impairment. The area under the curve (AUC) and mean residence time of MEL are increased in MM patients with renal dysfunction [32]. There is a significant correlation between CrCl and the pharmacokinetic parameters of MEL, indicating interindividual variation [33]. MM patients with chronic kidney disease (CKD; CrCl < 60 mL/min) experience higher rates of severe mucositis and other complications in comparison to those without CKD [34]. Therefore, the dose of MEL needs to be decreased to 140 mg/m^2^ in patients with CrCl 15–59 (mL/min). This dose seems to be as effective as a dose of 200 mg/m^2^ [35]. However, in this study, MEL dose adjustments were determined by considering not only the CrCl value but also the patient’s overall physical condition, hepatic and cardiac function, and other factors. Therefore, it is possible that elevation of the blood concentration of MEL in patients with renal impairment who received a dose of 200 mg/m^2^ may have led to CINV in the beyond delayed phase in this study.

In this study, nausea was assessed based on the percentage of patients who received TPN (i.e., grade 3). Relative to oral and enteral nutrition, the use of TPN is associated with reduced gastrointestinal integrity, increased risk of central venous catheter infection, and bacterial translocation [36]. The reduction in the proportion of TPN use in the IAR group may contribute to maintaining gastrointestinal function, reducing the risk of infection, and preventing the deterioration of the mucosal barrier function.

The incidence of grade 3 diarrhea was significantly lower in the IAR group, while the incidence of any-grade constipation was significantly higher in the IAR group. These effects may be attributed to additional doses of DEX and APR, and their drug-drug interaction [24]. APR inhibits CYP3A4 activity and reduces DEX clearance by approximately 50% [25] In this study, increased blood concentrations of DEX may have led to constipation as an adverse event and reduced the incidence of grade 3 diarrhea. Severe diarrhea in hospitalized patients can cause perianal skin damage, extend hospital stays due to *Clostridioides difficile* infection, and reduce patient QOL [37]. Therefore, reducing the occurrence of diarrhea should help mitigate the risk of infection and improve QOL in patients. The incidence of any-grade hiccup was significantly higher in the IAR group. However, all patients who experienced this recovered within 7 days, suggesting that the condition was manageable. There were no adverse events leading to treatment discontinuation in this study, indicating that the four-drug intensive regimen including OLA can be used safely.

This study had several limitations. First, this retrospective study compared patient populations before (standard group) and after (IAR group) April 2021. The multivariate analysis may have been unable to adjust for all confounding factors, although the standard treatment for MM patients and the treatment process including induction therapy and transplantation at the facility did not change. Secondly, we could not assess mild nausea. Consequently, we could not evaluate indicators such as “complete control” or “total control”. Third, adverse events such as somnolence, dry mouth, and dizziness, which might be associated with OLA, could not be evaluated. Fourth, it was not possible to collect patient background factors such as alcohol use, smoking history, morning sickness during pregnancy, or motion sickness, which are risk factors for vomiting.

## Conclusion

Our findings indicate that the four-drug intensive regimen including OLA may improve the antiemetic effect on delayed and beyond delayed CINV while also ensuring safety in Japanese MM patients undergoing MEL/ASCT.

## Data Availability

No datasets were generated or analysed during the current study.
